# ICPD to MDGs: Missing links and common grounds

**DOI:** 10.1186/1742-4755-5-4

**Published:** 2008-09-10

**Authors:** Farina G Abrejo, Babar T Shaikh, Sarah Saleem

**Affiliations:** 1 Health Systems Division, Department of Community Health Sciences, Aga Khan University, Karachi, Pakistan; 2 Population & Reproductive Health Program, Department of Community Health Sciences, Aga Khan University, Karachi, Pakistan

## Abstract

The ICPD agenda of reproductive health was declared as the most comprehensive one, which had actually broadened the spectrum of reproductive health and drove the states to embark upon initiatives to improve reproductive health status of their populations. However, like all other countries, Pakistan also seems to have shifted focus of its policies and programs towards achieving MDGs. As a result, concepts highlighted in the ICPD got dropped eventually. In spite of specific goals on maternal and child mortalities in MDGs and all the investment and policy shift, Pakistan has still one of the highest maternal mortality ratios among developing countries. Lack of synchronized efforts, sector wide approaches, inter-sectoral collaboration, and moreover, the unmet need for family planning, unsafe abortions, low literacy rate and dearth of women empowerment are the main reasons. Being a signatory of both of the international agendas (ICPD and MDGs), Pakistan needed to articulate its policies to keep the balance between the two agendas. There are, however, certainly some common grounds which have been experimented by various countries and we can learn lessons from those best practices. An inter-sectoral cooperation and sector wide approaches would be required to achieve such ambitious goals set out in ICPD-Program of Action while working towards MDGs. There is a need of increasing resource allocation, strengthening primary health care services and emergency obstetric care and motivating the human resource employed in health sector by good governance. These endeavors should lead to formulate evidence based national policies, reproductive health services which are affordable, accessible and culturally acceptable and finally a responsive health system.

## Introduction

The International Conference of Population and Development (ICPD) held in Cairo in 1994, presented a Program of Action (PoA) which had pledges to achieve the goal of universal access to reproductive health (RH) services for every one in all countries till 2015 [1,2]. In all, 179 countries became signatories and pledged to make change in their legislation and reproductive health (RH) related policies according to program of action. Five years later in 1999, a review of ICPD-PoA, known as ICPD+5 revealed that there was a need of sufficient domestic and external resources to be invested in order to achieve the goals of ICPD in next 15 years in many countries. In 2000, world leaders expressed that there is a need to have explicit goals to measure the pace of socio-economic development at country level all over the world. Among these goals, one explicit goal was on universal access to RH information and services, similar to the one described in ICPD-POA. In the same year, there was UN Millennium Summit where the transformation of International Development Goals (IDGs) into the Millennium Development goals (MDGs) took place. However, surprisingly only eight goals instead of nine were presented; the goal on the RH was dropped [3]. However, after lobbying by many governments, nongovernmental organizations and others, world leaders at the September 2005 UN World Summit in New York endorsed incorporating universal access to reproductive health into the MDGs. A comprehensive review of ICPD at the mid point to 2015 was recognized in 2004 as ICPD+10, which yet again discovered many countries lagging behind in RH indicator nor they are anyway near to the progress demonstrated in developing regions of the world [4,5].

Pakistan is the signatory of ICPD's PoA and MDGs both and the Government of Pakistan is geared to achieve the targets till 2015. Although, ICPD agenda sets a ground for all the countries to ensure the access to RH through primary health care (PHC), yet all the programs and the health care system in the country failed to ensure the provision of essential RH services such as RH education, family planning counseling, safe delivery services, post-natal and safe abortion services to the majority of women population in the country [6]. For addressing this alarming situation and dismal state of RH and women's health indicators in the country, it becomes imperative to identify "the missing links" between the ICPD agenda, government's policies, national programs, and the efforts launched to achieve MDGs. This paper explores the gaps and missing links in the journey from ICPD 1994 to MDGs 2001. The conclusions are drawn for identifying the commonalities between ICPD and millennium agendas other international treaties and government's policies, strategic plans and programs to suggest modalities to embark upon or scale up concrete initiatives for achieving MDGs.

### Methodology

Literature review was carried out for the period of 1994–2001, thus selecting the published literature on either ICPD or MDGs using Google, Medline and websites of various organizations. Key words used for the search included Millennium Development Goals; ICPD; Reproductive health; Developing Countries; and Pakistan. Peer reviewed articles, reports, strategy papers and official publications of government, UN agencies, World Health Organization, multilateral and international donors, NGOs and various MDGs monitoring agencies were consulted. For describing the scenario in the local context, a search period of 1994–2005 was determined in which official documents and reports of Government of Pakistan were reviewed.

## Results

### International response to reproductive health

For addressing the pace of population growth and related issues in developing countries, United Nations held a conference at Cairo in 1994. One of the outcomes of this conference was the call of new paradigm in reproductive health. This new paradigm increases the emphasis on human rights, human development and individual well-being which should be the center of all RH related programs and policies [2]. The key goals which were embedded in the ICPD-PoA were:

• By 2005, 60% of primary health care and family planning facilities should offer the widest achievable range of safe and effective family planning methods, essential obstetric care, prevention and management of reproductive tract infections, including sexually transmitted infections (STIs), and barrier methods to prevent infection; 80% of facilities should offer such services by 2010, and all should do so by 2015.

• Skilled attendants should assist at least 40% of all births where the maternal mortality rate is very high; and 80% globally by 2005. This coverage should be 50% and 85% by 2010; and 60% and 90% by 2015.

• The gap between the proportion of individuals using contraceptives and the proportion expressing a desire to space or limit their families should be reduced by half by 2005, by 75% by 2010, and by 100% by 2015 [2].

Among the 15 principles of PoA, it was clear that issues such as family planning, infant mortality and morbidity, maternal mortality and morbidity and sexually transmitted infections (STIs) have been placed in such context where these are sighted from the broader angle of RH for women and men of all ages. Empowerment of women through ensuring their ability to control their own fertility is the cornerstone of population and development-related programs. The right based approach is the major theme of ICPD inclusive of inclusive of STIs and prevention of HIV, human sexuality and gender relations, reproductive rights for adolescents, family planning. In pursuance, all the countries agreed to provide RH services accessible through primary health care system. After five years (in 1999), another session was held in which all the ICPD signatories and European Union got together and adopted a document named "Key actions for the further implementation of the Program of Action of ICPD". At this stage one particular addition was the inclusion of HIV/AIDS preventive services especially for pregnant women. The major focus of this conference was to how to overcome the identified barriers for achieving the goals set in Cairo particularly human and legal impediments in access of services. At least 20% of resources for RH programs were suggested to be earmarked for meeting the adolescents' information and services needs [7].

With the agenda to provide development in the economical, social and environmental domains, the international community adopted the International Development Goals (IDGs). Interestingly, one goal was exclusively on providing and improving access to RH services for all females of appropriate age [8]. In the year 2001, MDGs were introduced whereby RH was not included at all in the eight goals. Several reasons have been quoted so far among which most cited is the difficulty for the governments to sustain the commitment to broader rights issue, while facing the opposition around the world [9]. However, goals 4–6 still address RH directly i.e. maternal health, infant mortality and HIV/AIDS.

In 2004, the 37^th ^session of the United Nations Commission on Population and Development undertook a comprehensive review of progress in the last ten years (known as ICPD+10) on all aspects of the ICPD PoA. ICPD+10 acknowledged that full implementation of the Cairo agenda is essential to the attainment of Millennium Development Goals (MDGs), and that this link must be stressed at the five year review of the Millennium Declaration. The main barriers identified, however, remain unresolved. These include inadequacies in the healthcare systems, unclear impact of health sector reforms, lack of a national system for government reporting and accountability, negative impact of development institutions and donor policies and inadequate NGOs' policy advocacy capacity. Ascertaining the position assumed for various themes of ICPD in different international conference held after 1994 till MDGs in 2001 is presented in the Table 1.

**Table 1:** Global stance on reproductive health in international treaties

**ICPD 1994 PoA**	**ICPD+5 1999**	**ICPD+10 2004**	**Beijing Conf 1995**	**MDGs 2000**
Reproductive Rights	√	√	√	×
RH care services	√	√	√	×
Make RH services accessible through PHC	√	√	√	×
Educate adolescents	√	√	√	×
Community participation by decentralizing the management	√	√	×	×
RH services for Migrants	√	√	×	×
Family Planning Services	√	√	√	×
Abortion	√	√	√	×
Promote breast feeding	√	√	√	×
Involvement of NGOs	√	√	√	×
Institute system of monitoring & evaluation	√	√	√	×
Involvement of political & community leaders	√	√	√	×
Proper referral mechanism	√	√	√	×
Expand/upgrade training in RHC providers	√	√	√	×
Reducing maternal mortality and morbidity	√	√	×	√
Emphasis on donor support	√	√	×	×

### Translation of ICPD, program of action in pakistan

In 1950, Pakistan had a population of about 40 million people; today it is around 160 million [10]. According to National Health Survey of Pakistan (1990–94), it was lack of adequate RH services responsible for a high fertility rate and a high maternal mortality ratio in Pakistan. Most of the deliveries 80% were conducted by traditional birth attendants or the near-relatives [11]. Pakistan National Health Policy 1997 did reflect the holistic approach to address the issues of RH with the inclusion of rights through primary health care (PHC) as described in ICPD agenda. Despite the government's high level of commitment towards increasing the RH and women's health services, the health of the population suffered a setback due to frequent political changes, the structural adjustment program, inflation and lower social sector allocations. The momentum continued at the policy level under the Ninth Five Year Plan (1999–2003). A draft National RH Policy was proposed in 2000 using the ICPD definition of RH including the ensuring of reproductive rights and women's empowerment for participation in "all aspects of reproductive decision making on a basis of equality with men" [12]. The policy, however, was never formally approved. On the other hand, progress in term of reaching major targets has been painfully slow [13]. Some of the highest rates of under 5 mortality and infant mortality are found in Pakistan [14]. While there is little population-based information on maternal mortality, available data indicate that maternal mortality rates are unacceptably high and also intransigent to change [15]. Among others the main challenge is low access (both in terms of availability and affordability) to good quality nutrition and poor management of health care and childhood illnesses. One explanation is inadequate public health expenditure in face of ever increasing demand. According to a report by WHO Commission on Macroeconomics and Health, US$34 per capita is required for a package of essential health services in Pakistan The total expenditure on health in Pakistan is US$18 per capita out of which the total government health expenditure is US$4 per capita, which falls drastically short of the recommended level [16]. Pakistan's lack of effort in allocating public resources where needs are high also applies to the health sector. Yet, Pakistan's public expenditure on health stood at just 0.57% of GDP in 2007, the second lowest of the 29 countries of Asia Pacific [17]. The government of Pakistan has been spending 0.6 to 1.19% of its GDP and 5.1 to 11.6% of its development expenditure on health over the last 10 years, more than 45% of this meager budget would be consumed by curative services, mostly at tertiary hospitals [18]. Due to poor quality and unavailability of basic health care services, the RH of the people is also getting worse. According to Pakistan Demographic and Health Survey (PDHS) 2006–2007, the Total Fertility Rate (TFR) is 4.1 (which was 4.3 in the year 2000) and the contraceptive prevalence rate (CPR) is lower than ever (30 percent), which shows that the country is not on track neither for the MDGs nor for the ICPD. In spite of having a vast infrastructure, the PHC network is under-utilized and provides limited services to the rural and peri-urban populations [19,20]. A recent survey indicates that, nationally, only 20.6% of the people used the public sector network for their health care needs [21].

### Other national policies, programs and projects articulating RH agenda

In 1997, government revised and updated the first ***National Health Policy of 1990***, including the specific objective of "expending the delivery of RH services including family planning both in urban and rural areas of Pakistan". At this period of time (1997), there were 4,250 Lady Health Visitors (LHVs) and 50,000 trained birth attendants (TBAs). On the other hand, there were geographical imbalances in the distribution of health facilities and manpower in rural and urban areas. The ***National Health Policy of 1997 ***were based on the goal "Health for all" through PHC, therefore the vision for the health sector development was made for the year 2010. For prevention and treating common ailment at the community level, ***the Prime Ministers Program for Family Planning and Primary health care ***was started in 1994. There are 100,000 lady health workers (LHW) working in this program with a system of supervision and monitoring of Lady Health Supervisor looking after 20–25 LHWs. In this program, one specific goal is on expanding the family planning services availability in urban slums and rural areas of Pakistan. However, main purpose of the program is to bridge the gap between the community and health services through referral system [22,23]. In the year 1999, the ***RH Service Package ***was developed with the joint effort of Ministries of Health and Population Welfare. For providing broad guidelines, eight components of RH were included in this package; comprehensive family planning, maternal health care and safe motherhood, pre and post abortion care, infant health care, prevention of STDs and HIV, management of infertility, detection of breast and cervical cancers, and management of RH related problems of men. This package was aimed to impart all the skills to TBAs, midwives, Lady Health Workers, Lady Health Visitors, health technicians at the Basic Health Units//Rural Health Centers, lady doctors and male doctors. In addition, the package also provides a complete framework for involving NGOs [24]. Thereafter, a draft ***RH Policy ***was also presented in 2000 based on the ICPD's PoA but was never approved by Ministry of Health or Population welfare [12]. In the year 2001, the ***National Health Policy (NHP) ***was presented with overall national vision for the health sector based on "Health-For-All" approach. The NHP had ten key areas, among which key area 4 somehow explains the need for gender equity in the health sector focusing on RH services provision to the women of childbearing age at the doorsteps [25]. In the same year, ***Population Policy ***of Pakistan was also launched, which explicitly identified the issues related to increasing population of the country. Fortunately, this policy did carry the core mission of ICPD in its vision, "to achieve population stabilization by 2020 through the expeditious completion of the demographic transition that entails declines both in fertility and mortality rates" [26]. With the parallel goals of 'eliminating poverty' and 'ensuring gender equity', recognized as essential for achieving RH agenda, ***National Policy for Development and Empowerment of Women ***was introduced in 2002 which reflected government's yet another reaffirmation on 'women and girls access to quality health care services and all other pre-requisites to enjoying full health, including reproductive and mental health'. Issues such as RH rights, strengthening of basic health facilities, providing affordable and preventive primary health care particularly RH services for the women were addressed in this policy [27]. Later on, when many developing countries adopted the ***Poverty Reduction Strategy Paper *(PRSP) **with explicit strategies linking MDGs with poverty reduction initiatives, Pakistan introduced its PRSP in 2003. This paper clearly stated that the medium term health strategy is focused towards raising public sector health expenditures through a focus on prevention and control of disease, RH, child health and nutrient deficiencies. In this context, the number of LHWs is increased to 100,000 to provide the PHC at the doorstep through an integrated community based approach [28]. Though, RH inclusion is restricted only to some explicit services, the government's intention, however, has attracted some donors' assistance for maternal and child health projects. The government currently is revising the PRSP which again do not reflect RH promotion agenda of ICPD [29]. Additional file 1 shows the position of various RH constituents across different policies and strategy papers of government of Pakistan.

## Discussion

The ICPD agenda has been declared as the most comprehensive programme including the goals and targets, which has actually broadened the spectrum of reproductive health. It was a complete paradigm shift for those, who always limited RH to family planning. While the MDGs introduced later were merely indicators to monitor the progress and moreover completely missed a goal encompassing RH agenda of ICPD. Therefore, RH is considered as a 'missing link' for MDGs, despite the fact that some quarters still believe that the essence of RH is present in goal 4 (child mortality), goal 5 (maternal mortality) and goal 6 (HIV/AIDS, Malaria and tuberculosis). If at all we agree that the complex RH agenda of ICPD is present in MDGs, the broader issue of rights which was stitched with RH is clearly dropped in MDGs. The maternal mortality reduction goal, for example, cannot be achieved without access to the full range of reproductive health services, and without empowerment of women. Women need a stronger voice, and there is a need for much stronger reflection of women's own health needs in prioritization of health services.

In the context of Pakistan government's policies, all efforts had been geared to achieve the targets of MDGs. Hitherto, the policies and programs started after MDGs in Pakistan have missed the true RH agenda altogether. Therefore, the concept of reproductive rights has not been much visible in the policies and the process of women empowerment could never gain real momentum in Pakistan except in last few years. All the programs and projects seldom supported RH issues with rights approach. Lot of focus and investment on maternal and child health programs is yet to deliver results because maternal mortality ratio (MMR) in Pakistan is still one of the highest in the region than the rest of the developing world [30]. Maternal mortality ratio is alarmingly high in Pakistan, gone up from 350 per 100,000 live births in 2000–01 to 400 per 100,000 live births in 2004–05 [31]. The issue of maternal mortality highlighted in ICPD agenda was linked with unskilled birth attendants. According to a survey done in 2003, only 44% of recent mothers received antenatal care and only 28% delivered in health care facility [19]. Another reason which is highlighted through the literature review for frightening maternal mortality ratio is increasing number of unsafe abortions. Abortion has always been inadequately addressed in all of policies (see additional file 1) because it has always been considered a taboo and made a religious issue. So far, very few studies have been conducted in the country for knowing the burden contributed by unsafe/illegal abortion to the maternal mortality. National study on abortion produced by the Population Council revealed high level of unwanted pregnancy, induced abortion and post-abortion complications [32]. Despite living in the domain of Islam, many countries found workable strategies for this problem by giving it a different position [33,34]. Another linked issue is the inadequate family planning services which also increase the number of illegal abortions in the country. Lady Health Workers (LHWs) were always considered as the key players in providing FP services at the doorsteps in the communities. Yet after fifteen years, the program has not shown its expected impact; the contraceptive prevalence rate (CPR) is as low as 28–30% [31]. Independent evaluations of LHW program pointed out the difficulty faced by LHWs in reaching out to vulnerable populations in the remotest and conservative parts of the country. According to ICPD, there is a need of increasing constructive collaboration among NGOs and government for providing accessible and affordable services of RH to population. NGOs actually helped promoting the services of RH in the country. Despite clear emphasis in ICPD agenda for increasing the donors' contribution to the countries to provide funds to improve their RH indicators [35], it did not virtually happene due to continuous changes in donor preferences after MDGs. Among other reasons, the Global Gag Rule consistently reduced the funds for RH programs and projects in many developing countries [36]. The case is not different for Pakistan, where many international donor agencies are working but the focus is more towards the programs addressing MDGs on AIDS, TB, Malaria, etc [37].

**Figure 1:**
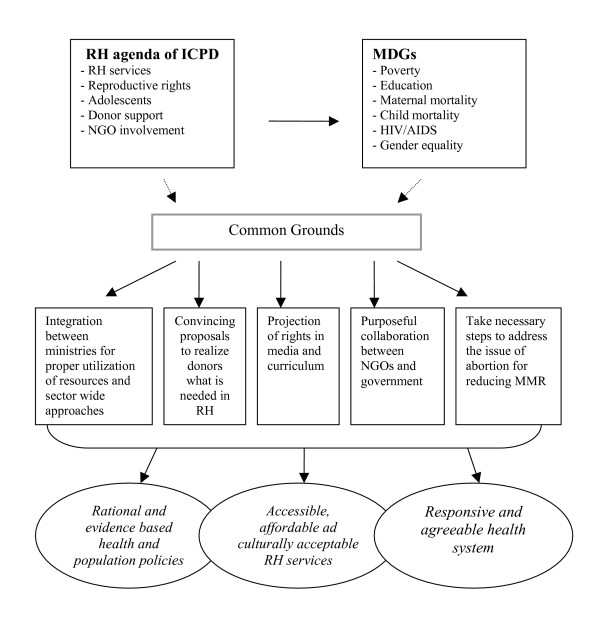
Expected outcomes of working on common grounds between ICPD and MDGs.

Several countries are using various frameworks and processes, such as PRSPs, sector wide approaches (SWAps) and administrative reforms to promote the ICPD agenda through international cooperation with donors. For instance, in Canada, the Canadian International Development Agency (CIDA) cooperates with its partners and concerned stakeholders to categorize and promote ICPD-driven priorities, working within national poverty strategies and sector reforms. In Japan, the government employed SWAps and PRSPs proactively and provided support for basic data collection for poverty analysis and for enhancing national capacities to collect, analyze and publicize basic data for implementation of the ICPD PoA. In Norway, the MDGs are used to set priorities and as a reference in the dialogue with development partners to emphasize the significance of addressing such issues as maternal health and the prevention of HIV/AIDS. Sweden also promotes the ICPD and ICPD+5 agenda through PRSPs, SWAps and sector reforms and through constant dialogue with governments and stakeholders participating in programmes pertaining to sexual and RH and rights; the needs of young people; gender equality and the role of men; and HIV/AIDS prevention, care and support. In Switzerland, the Swiss Agency for Development and Cooperation (SDC) has built up its policies, strategies and guidelines aimed at contributing to the achievement of the MDGs and ICPD goals. For instance, its new health policy aims to improve the health of the underprivileged and most vulnerable populations in order to supplement the efforts for poverty reduction and sustainable development. Similarly, in United Kingdom, the Department for International Development (DFID) is advocating the use of PRSP and budgetary support mechanisms based on the priorities expressed by developing countries themselves and where key MDGs and RH indicators are used to follow the progress. In the United States, the Government is placing increased accent on poverty reduction. A 'Millennium Challenge Account' has been created, whereby funds are allocated for poverty-reduction efforts in some partner countries [38]. Some of the expected outcomes of working on common grounds between ICPD and MDGs are hence shown in figure 1.

## Conclusion

Besides continuous efforts of the international RH community, the donor preference for investing in RH programs did not change much particularly for developing countries like Pakistan. Now after years of inception of MDGs, many countries are still not on track to progress to achieve the targets set out in 2001 and Pakistan is one of these countries lagging behind with maternal and child mortalities rates higher than many developing counties [39]. The half way point MDGs monitoring report of the government narrates that MDG 5 on maternal health is one of the most important but most difficult to achieve of the eight goals. The government is committed to work with NGOs and the private sector to complement the information and services that it is itself providing [31].

Pakistan has many policies, programs and projects for giving a proper place to the goals of MDGs. For instance, National Maternal and Child Health Policy put a great deal of emphasis on raising awareness about safe motherhood, newborn's health, family planning and increasing number of skilled human resource. Still the program is not getting the desired outcomes [40]. Being a signatory of both of the international agendas (ICPD and MDGs), Pakistan needed to articulate its policies to keep the balance between the two agendas which is, however, not visible. The national policies are deficient in addressing the common RH problems (adolescent RH, abortion, referral mechanisms etc) and on the other hand, the entire emphasis seems to be on promoting family planning alone. There are, however, certainly some common grounds which have been experimented by various countries and we can learn lessons from those best practices. An inter-sectoral approach and SWAps would be required to achieve such ambitious goals set out in ICPD-PoA while working towards MDGs. An approach which would necessitate involvement of NGOs, donors and other stakeholders from the civil society for launching combined efforts to address a complex horde of issues around RH [41]. The Commission on Macroeconomics and Health recommends increasing resource allocation to education and health; deploying more female health providers in rural areas; strengthening primary health care services and Emergency Obstetric Care (EmOC); and initiating meaning reforms which are capable of elevating the social status of women [16]. Government's commitment has to be reiterated in terms of strengthening and expanding the promising LHW program by attracting more donor support and raising the workers incentives. These endeavors should lead to formulate evidence based national policies, RH services which are affordable, accessible and culturally acceptable and finally a responsive health system. Poverty reduction strategies, reforms in education and health sector and planning for 2015 to achieve MDGs could be actually harmonized to deliver desired outcomes that were set in ICPD 1994. This would, however, necessitate sector wide approaches for designing various programs, building confidence of donor community, believing in NGOs credibility and overall strong political will to improve RH and especially women's health indicators of under-served segments of population in the country.

## Competing interests

The authors declare that they have no competing interests.

## Authors' contributions

FGA made substantial contributions to conception and design, acquisition of data, analysis and interpretation of data. BTS supervised the concept and design, helped in literature search and synthesis, and contributed in producing drafts. SS supervised data compilation and production of all the drafts. All authors read and approved the final manuscript.

## Supplementary Material

Additional file 1 Table 2. National policies showing the position of various reproductive health components. Click here for file
