# Supplementary material for: ICPD to MDGs: Missing links and common grounds
Source: Reprod Health. 2008 Sep 10;5:4. doi: 10.1186/1742-4755-5-4 (PMC2546384; doi:10.1186/1742-4755-5-4)
Supplement: Additional file 1 — Table 2. National policies showing the position of various reproductive health components. [file 1742-4755-5-4-S1.doc]

**Table 2**: National policies showing the position of various Reproductive Health components.

| **ICPD 1995 PoA** | **National Health Policy 1997** | **RH Policy (draft) 2000** | **RH Package**  **1999** | **National Health Policy 2001** | **Population Policy 2001** | **PRSP 2001** | **Women Development & Empowerment policy 2002** | **PC-1 MoPW** | **MTDF**  **1. Health**  **2.Population Welfare** |
| --- | --- | --- | --- | --- | --- | --- | --- | --- | --- |
| Reproductive  Rights | × | **√** | × | × | × | × | √ | × | 1. × 2. √ |
| RH care services | √ | **√** | **√** | **√** | **√** | **√** | √ | **√** | 1. × 2. √ |
| Make RH services accessible through PHC system | √ | × | **√** | **√** | × | **√** | √ | × | 1. √ 2. √ |
| Educate adolescents | × | **√** | **√** | × | × | × | √ | √ | 1. × 2. √ |
| Community participation by decentralizing the management | √ | **√** | × | × | × | × | × | √ | 1. × 2. √ |
| RH services for Migrants | × | × | × | × | × | × | × | × | 1. × 2. × |
| FP Services | √ | √ | √ | √ | √ | × | √ | √ | 1. √ 2. √ |
| Abortion | × | × | √ | × | × | × | × | × | 1. × 2. × |
| Promote breast feeding | √ |  | √ | × | × | × | × | √ | 1. × 2. × |
| Involvement of NGOs | √ | √ | √ | × | √ | √ | √ | √ | 1. **×** 2. √ |
| To institute system of monitoring and evaluation | √ | √ | √ | × | √ | √ | √ | √ | 1. √ 2. √ |
| Involvement of Political and community leaders | √ | √ | × | × | **√** | × | √ | **√** | 1. × 2. √ |
| Proper referral mechanism | √ | × | √ | × | **√** | **√** | √ | **√** | 1. × 2. √ |
| Expand/upgrade training in RHC providers | √ | × | √ | × | **√** | **√** | √ | **√** | 1. × 2. √ |
